# Relational practice, a critical component for successful social work

**DOI:** 10.1080/23311886.2025.2492402

**Published:** 2025-04-24

**Authors:** Katy Cleece, Bethany Cheneler, Joanna Harrison, James Hill

**Affiliations:** Lancashire and South Cumbria NHS Foundation Trust; School of Medicine and Dentistry, University of Central Lancashire; Synthesis Economic Evaluation and Decision Science Group (SEEDS), University of Central Lancashire

**Keywords:** relational practice, relationship, trust, social work

## Abstract

Relational practice describes the value and development of relationships or connections with others and is a key concept across the different systems of health, education, criminal justice, and social work. Lamph et al. (2023) undertook a scoping review of the literature to support a shared understanding of ‘relational practice’ in organisational and systemic contexts, noting impacts and benefits reported. They identified that relational practice may be beneficial to both the service users and the workforce across different sectors. The review authors also found that variations exist in the terminology used for ‘relational practice.’ This commentary describes the importance of relational practice for social work, building on the review by Lamph et al. (2023) and the implications of the findings for social work practice and research. Relational practice is pivotal to social work and is vital for meaningful intervention, yet there is a need for more scientific effectiveness studies, if relational practice is to receive the recognition it deserves, and for it to become fully embedded within different sectors.

## Introduction

Globally, social work is defined as a practice-based profession, underpinned by academic theory, supporting the liberation and empowerment of people to achieve social change, social cohesion and founded in principles of social justice, human rights, collective responsibility and respect for diversities (IFSW 2014). Registration to practice social work in the UK is dependent on adherence to practice guidance set by the Department of Health and the British Association of Social Work (BASW 2018, DoH 2015). Each place significance on the quality of the relationship with the individuals accessing social work, the intervention, the authority social workers hold and their responsibility to acknowledge and address the power differentials within the relationship (BASW 2018, DoH 2015).

The ability and commitment to build safe and trusting relationships with people who are vulnerable, have history of abuse or maltreatment, or are fearful and rejecting of intervention is a skill pivotal to social work, and reducing harmful outcomes (Frederick, Spratt and Devaney 2023, Healey 2015, Ingram and Smith 2018, Rollins 2019). For social workers, relationships are key to working collaboratively with individuals and families to share understanding of what needs to be done and by whom (Ruch 2020). Recognising a person’s history of trauma and applying trauma informed approaches helps to establish trusting relationships, vital for meaningful social work intervention (Levensen 2017). Conversely, the absence of trust in the relationship with a social worker makes meaningful involvement less likely (Cossar, Brandon and Jordan 2016). It should be highlighted that in the instance of high-profile serious case reviews of abuse, that have received much public attention, such as the Daniel Pelka and Victoria Climbié cases, relationship-based practice appears to have been lacking, with the case reviews identifying insufficient efforts from social care staff to forge trusting relationships directly with the children in concern (CSCB 2014, RBSCB 2013, Rustin 2004).

Hence the imperative need for relational practice in social work. As a construct, Relational practice has many component parts, an integral one being the ability to reflect, and in doing so evidence self-awareness of how one relates to and responds to service users. (DfE 2020, Ruch 2020). It recognises the balance of power between worker and client and utilises a more personal and intuitive approach, such as employing the attributes of helping and compassion rather than compliance and intervention. (Ingram and Smith 2018). Effective relationship-based and reflective practice requires vison, understanding, commitment and perseverance, and should be embedded in social work organisations from top to bottom (Ruch 2020).

A scoping review by Lamph et al. (2023) aimed to interpret how relational practice is used, defined, and understood with a focus on organisational and systemic practice, across health, criminal justice, education and social work, including reported impacts and benefits. The authors defined relational practice within these contexts as a practice and/or intervention that prioritises interpersonal relationships in service provision for both external (organisational contexts) and internal (service user/worker experience) aspects. Given the significance of relationship-based practice for the social work profession, it is timely and relevant to critically review and explore Lamph et al. 2023 in this context. This commentary will critically appraise the methods used by Lamph et al. 2023, and discuss the findings, with a specific focus on social work including the implications for social work practice, training and future research.

### Findings of Lamph et al (2023)

From 11,490 articles initially identified, 521 remained for full-text review and 158 were included in the synthesis. Most of the included articles were from the UK (30%), USA (20%) and Canada (16%) but included a broad and global spread of literature. Most literature came from the health sector (38%) followed by education (26%), social work (25%) and criminal justice (11%). Most included papers were theoretical, or opinion based (39%), reported qualitative findings (28%) or were case studies (17%). A small number of included studies used a quantitative design (4%) and 6% were mixed methods. A further 4% were narrative reviews, and 2.5% systematic or scoping reviews.

Across the different organisational sectors, there were commonalities in the terms used to define relational practice, but equally different terms were used for specific sectors. For social work/care, terms were more unique to relational social work practices such as relational theory or practice, relationship-based practice, guanxi (Chinese term meaning relationships), family centred inclusive practice, relational authority and strength, restorative practice.

Seventy-six articles (48%) reported impacts or benefits of relational practice. Of these, workforce or client impacts were the most reported (54%) with the benefits of this way of working including enhanced knowledge, insights, healthy working environments, enhanced team cohesion, shared experiences and understanding of interpersonal dynamics. Some articles also reported enhancement of interpersonal skills including communication and empathetic listening. Other gains included personal benefits such as those relating to confidence, increased employee satisfaction and making more of an impact for clients in terms of their progression and achievements.

In terms of client health, impacts were found in relation to enhancement of well-being, physical, psychological, and social impacts, and various educational attainments. Recovery from client difficulties including substance misuse and improved child custody were also reported, alongside enhanced interpersonal relationships with service providers and carers/families. Further health impacts included reductions in trauma and re-traumatisation, emotional regulation skills, and buffering of stress. There were also reductions in re-offending and violent incidents in criminal justice contexts. Other positive impacts included a reduction of health inequalities, engagement with society and community belonging. Where relational practices were not used, it was identified that promoting a sense of belonging could be overlooked.

Organisational impacts were less frequently reported but referred to poignant and important learning, including the development of healthy sustainable communities. Relationship and interpersonal work were often described as invisible but crucial to working with people facing services as well as the importance of collaboration. Relational practice was also valued for its potential to create an environment with a focus on interagency working and emotional availability, enhancing the wellbeing of both clients and the workforce.

### Critical Appraisal of the Review by Lamph et al. (2023)

Using the Joanna Briggs Institute checklist for systematic reviews and research syntheses (JBI 2024), the scoping review achieved all relevant criteria (see Table 1.). Three criteria were non-applicable primarily due to the nature of scoping reviews (critical appraisal of primary studies is non-essential [Peters et al. 2020], and no assessment of publication bias due to primary studies being of a qualitative nature). Therefore, based on the JBI checklist, the scoping review can be deemed to provide a comprehensive synthesis or mapping of the extant relational practice literature. It is worth noting that the review authors acknowledge the focus of the review on organisational rather than individual practice as a limitation. However, it is suggested that this is in response to a gap in the literature for reviews that focus on organisational practice, and also to feasibly conduct the review within existing resources.

### Discussion: Implications for practice

#### Workplace benefits of relational practice

The Professional Capabilities Framework (PCF), an overarching framework of social work education and professional development in England describes knowledge from social work practice and research, and people who use services, as key domains of social work practice that social workers are expected to develop (BASW 2018). The findings from Lamph et al. (2023) suggest that using relational practice may enhance knowledge, insights and understanding of interpersonal dynamics, helping to meet these expectations. This could include being culturally competent, an umbrella term that revolves around the principles of self-awareness, acceptance, and the ability to adapt and learn from colleagues' individual lived experiences (Ferdman and Deane 2013). This in turn helps to enhance shared experiences and a cohesive workforce, which Lamph et al. (2023) comment upon, with evidence suggesting that effective teamwork in healthcare is associated with job satisfaction, improved worker outcomes, staff retention and improved clinical practice. (West and Lyubovnikova 2013). This is even more important given the current climate where social workers are struggling to cope with challenging caseloads, mounting pressures including the impact of the pandemic, longer hours, significant levels of chronic stress and poor staff retention rates (Beer and Asthana n.d, Curtis et al. 2010, Kinman and Grant 2017, Preston 2022), compromising the continuity and quality of service provision.

Engaging in relational practice therefore, can be mutually beneficial for both social worker and service user. Using the client's lived experience to guide and inform practice enables social workers to recognise and utilise their own lived experience, and navigate between their personal and professional boundaries, with a recognition of how prioritising connection over separation can contribute positively to their practice (O’Leary et al. 2013). It is also worth considering however the emotional toil and risks for social workers where organisational culture does not align well with relational practice. Social workers risk burnout or vicarious trauma if not adequately supported in highly emotive relationship based work. Dwindling resources often results in task driven practice with individual worker supervision often being the first to become neglected, risking both the health and psychological wellbeing of the practitioner, but also the quality of practice offered to the service user (Ravalier et al, 2023).

#### Client benefits of relational practice

Relationships are central to successful outcomes in social work (Trevithick 2003, Ingram and Smith 2018). Therapeutic change can be achieved via helpful relationships being developed and promoted by social workers immersing themselves in the physical lives of service users. This can be termed a “holding relationship” as social workers provide reliability and are emotionally close, but simultaneously critical, whilst being aware of power imbalances, to evoke positive change (Ferguson et al. 2020). The review by Lamph et al. (2023) found that by shifting from coercive controlling environments to those that are engaging through negotiation was key to success and that by working in this manner improvements could be made to client health, recovery, and avoid retraumatising clients. This is particularly pertinent as service users frequently have social work intervention imposed onto them, which often occurs at a time of trauma in their lives, creating mutual mistrust and poorer outcomes (Mason, Taggart and Broadhurst 2020).

The review found that positive engagement via enhanced interpersonal relationships saw improvements spanning client’s lives and contributed to reducing health inequalities, plus people developing a stronger sense of belonging to their communities (Lamph et al. 2023). Buchanan et al. (2023) identified that social support was the most protective factor following on from childhood adversity. This is best achieved where the organisation fosters a culture to create positive relationship building, with allocation of sufficient time, perseverance and patience to build and sustain relationships. (HM Inspectorate of Probation 2023). Furthermore, the impact of programmed interventions is secondary to the relationship between social worker and service user (Nicholson and Artze 2003). The scoping review by Lamph et al. (2023) asserts that an approach which centralises positive human relations at a systems level is relevant and transferable across a variety of settings that provide care, support or education.

A further key finding from the review was focussed on utilising interpersonal skills including communication and empathetic listening, both fundamental to achieving trusting relationships. This in turn fosters engagement within communities, a sense of permanence and sustainable change, and a reduction of further social work intervention. Social work is designed to improve the lives of individuals and families via addressing challenges they face, and it is increasingly becoming clear that relationship-based practice is a critical component of positive outcomes in social work, particularly for the service user. For example, adverse childhood experiences are widely understood to impact on the life course and require some specialist intervention yet the presence of a relationship with a trusted adult has reparative qualities, and social workers are well placed to be that trusted adult or provide connection for child and trusted adult (Frederick, Spratt and Devaney 2023).

Social workers are privileged advocates for marginalised groups in society who often suffer social exclusion due to the complexities of their circumstances (Craig, 2002) and the complex interplay often operating between service users’ personal situations and their broader socio-economic environment. Developing relational and communication skills helps empower social workers to engage and understand stakeholders, drive social action and advocate for systemic change (CSR Education 2024). Relational practice therefore provides an ideal vein by which to address social injustice as it imbibes the principles of communicating in a nuanced, non-judgemental, and unobtrusive manner (Ruch, 2005), encouraging confidence in the service user and providing them with the skills of self-advocacy to enhance their social situation, and thereby reduce the often-perpetual cycle of oppression.

This isn’t a simple feat to navigate however, as complex and sensitive cases can create an environment whereby professionals have to employ their professional boundaries in order to protect both theirs and the service user’s safety (Doel et al. 2010). This can strengthen the separation from clients that relational practice strives to break down (O’Leary, 2013), so emphasising the need for extensive training, mentoring, supervision, and reflexivity of social workers. Whilst the separation is always likely to exist to some extent, relational practice provides the tools to make it a softer and lesser barrier than it currently stands which can only serve to address social injustice.

#### Organisational benefits of relational practice

Relational practice was highlighted by Lamph et al. (2023) for its potential to create an environment with a focus on interagency working. Strong interagency working in addition to effective management oversight has previously been identified to overcome challenges and deliver reforms in an impressively efficient manner within the context of family safeguarding (Baxter et al. 2023). Lamph et al. (2023) also identified that practising with a relational approach, could enhance employee satisfaction; this being a positive asset. It is recognised by Tugade and Fredrickson (2004), that positive emotions, assist in building resilience, which is of key focus in the Social Work Organisational Resilience Diagnostic (SWORD) change project. This project supports the creation of a resilient and sustainable climate in social work organisations by utilising an accessible, research-informed diagnostic tool and workbook to understand, build and sustain resilience (Grant et al. 2022).

#### Further implications for training

The review by Lamph et al. (2023) recognises that social workers entering the profession need to learn more than just classroom-based education. The current UK degree programme incorporates a blend of academia and placement experience, supported by practice education, and underpinned by the Professional Capabilities Framework (BASW 2018). There is evidence to suggest that supporting experiential education, led by people who currently or have previously accessed services as a pre-curser to progressing through the social work programme, can play a powerful and insightful role in assessing students’ readiness to practice and embed the skills and traits which promote relational practice (Skilton 2011). Similarly, support for reflective practices and relationship-based approaches through supervision, peer support and the organisation were significant contributors (Russ, Lonne and Lynch 2020) reflective supervision may help ensure that a therapeutic approach is provided in practice, as this style of supervision encourages emotions to be explored and tools elicited to address challenging emotions. This in turn leads to greater resilience and an increased emotional reserve that is required when providing effective relational practice (Russ, Lonne and Lynch 2020). Reflective supervision requires a supervisor to facilitate conversations about challenging events experienced in practice, and to encourage supervisee reflection on these events to gauge how self-aware the supervisee is and therefore how well they are demonstrating the skills and characteristics for effective relational practice. (Calvert et al. 2017). Also, the use of a “buddy system” as opposed to a more formal supervision arrangement may be both cost and time saving long term, by identifying any issues in practice quickly as opposed to waiting for a formal scheduled supervision by which time the relationship between social worker and service user may have been negatively impacted.

#### Implications for future research

The review by Lamph et al. (2023) concludes that the scientific evidence base for relational practice is still lacking. Challenges lie in measuring and evidencing the impact of relational practice on service users, due to its delivery being comprised of so many differing values and attributes. The review highlights with concern that there is no standardised definition of relational practice, which means there is no common agreement upon the aspects it encompasses. This makes it a challenge to find a research tool that encompasses all the aspects that contribute to an impact. The authors note the considerable focus of relational practice on the professional having a strong self-awareness and reflective capacity, and reflexivity towards the client’s emotional responses and consider them as important values in driving forward a standardised definition. Therefore, as it is known that a continuous reflective structure may be utilised to reflect upon one’s relational practice (Ornstein and Ganzer 2005), it is suggested that the Reflective Practice Questionnaire (RPQ) be utilised in research to provide a self-report measure of reflective practice. It is based on four domains: reflection in action, reflection on action, reflection with others, and self-awareness. (Rogers et al. 2024). The questionnaire can be taken by a social worker at any stage in their career but is most pertinent to those in training or newly qualified stages to support development of the reflective skills needed to demonstrate relational practice (Rogers et al. 2024). The questionnaire scores can then be analysed to assess the level of social worker engagement with reflective practice and the extent of their self-awareness. Smith (2009) highlight the imperative need to involve service users in social work, so the service user’s perspective could be obtained by adapting the ‘Patient Evaluation of Emotional Care during Hospitalisation’ (PEECH) survey tool to a social care context, which would enable review of the relational aspects of a service users experience. (Murrells et al. 2013). By combining the PEECH survey tool and the Reflective Practice Questionnaire it may become possible for researchers to explore an association between a social worker having a high reflective capacity and an improved service user experience. This level of scientific study is now needed in future research to evidence the effectiveness of relational practice, for it to be fully adopted and supported across the sector going forwards.

## Conclusion

Using relational practice in social work has a beneficial impact for social worker, client and organisation. For social workers, enhanced knowledge and understanding of interpersonal dynamics can lead to increased employee satisfaction and confidence. This is of increasing importance due to challenging caseloads and increasing pressures. For client health, enhanced inter-personal relationships with service providers supports engagement, therapeutic change, and improved physical, psychological and social wellbeing. Organisational benefits relate to the importance of relational practice learning for inter-agency working and people-facing services. Organisational support for reflective training through supervision and peer support is important for effective relational practice. Future research could utilise measures of reflective practice experience for both social workers and clients, to further evidence effectiveness in social work.

## Figures and Tables

**Table 1:** JBI Critical Appraisal of Lamph et al. 2023 using the JBI Checklist for systematic reviews and research syntheses (JBI 2024).

## Data Availability

Data sharing is not applicable to this article as no new data were created or analysed in this study.
